# Understanding cardiac events in breast cancer (UCARE): pilot cardio-oncology assessment and surveillance pathway for breast cancer patients

**DOI:** 10.1007/s10549-024-07322-w

**Published:** 2024-06-26

**Authors:** Michael Cronin, Aoife Lowery, Veronica McInerney, William Wijns, Michael Kerin, Maccon Keane, Silvie Blazkova, Dina Neiuroukh, Michael Martin, Osama Soliman

**Affiliations:** 1 University of Galway, School of Medicine, Galway, Republic of Ireland; 2 Sligo University Hospital, Sligo, Ireland; 3 CORRIB Research Centre for Advanced Imaging & Core Lab, University of Galway, Galway, H91 V4AY Ireland

**Keywords:** Cardio-oncology, Breast cancer, Pathway, CTRCD

## Abstract

**Purpose:**

In Ireland, over 3000 patients are diagnosed with breast cancer annually, and 1 in 9 Irish women will be diagnosed with breast cancer in their lifetime. There is evidence that female breast cancer survivors are more likely to die of cardiovascular disease than their age-matched counterparts. Specific services for cancer patients suffering from cancer therapy related cardiovascular toxicity have led to a higher incidence of safe anti-cancer treatment completion. Such services are not widely available in our jurisdiction, and the purpose of this trial is to remedy this situation.

**Methods:**

This protocol describes a prospective, single arm, pilot feasibility study implementing a dedicated Cardio-Oncology assessment and surveillance pathway for patients receiving multimodal breast cancer treatment. It incorporates novel biomarker and radiomic surveillance and monitoring approaches for cancer-therapy related cardiac dysfunction into routine care for breast cancer patients undergoing adjuvant systemic chemotherapy.

**Results:**

Declaration of results will via peer reviewed academic journals, and communicated directly to key knowledge users both nationally and internationally. This engagement will be critical to enable to healthcare services and policy sector make informed decisions or valuable changes to clinical practice, expenditure and/or systems development to support specialized Cardio-Oncology clinical pathways. All data is to be made available upon request.

**Conclusion:**

Dedicated cardio-oncology services have been recommended in recent literature to improve patient outcomes. Our protocol describes a feasibility study into the provision of such services for breast cancer.

**Supplementary Information:**

The online version contains supplementary material available at 10.1007/s10549-024-07322-w.

## Introduction

Breast cancer is the commonest female malignancy in Europe [1]. Each year more than 2 million women are diagnosed with breast cancer globally with about 685,000 deaths from the disease in 2020 [2]. In Ireland, over 3000 patients are diagnosed annually, and 1 in 9 Irish women will be diagnosed with breast cancer in their lifetime [3]. Multimodal oncological and surgical practice has improved outcomes, making breast cancer highly curable if diagnosed and treated appropriately at an early stage [4]. Data from the National Cancer Registry confirm a decline in age-standardised mortality rates for breast cancer and a marked improvement in 5- and 10-year survival rates over the past 2 decades [3]. These improvements in breast cancer outcomes are due to earlier diagnosis with the introduction of screening programmes and therapeutic advances informed by an improved understanding of breast cancer molecular biology [3, 5].

As breast cancer-specific mortality improves, there is an increasing recognition of both other causes of mortality and unmet survivorship needs in patients living with and after breast cancer treatment. There is evidence that female breast cancer survivors are more likely to die of cardiovascular disease than their age-matched counterparts [6]. This relates not only to a higher prevalence of risk factors for cardiovascular disease such as dyslipidaemia, abdominal adiposity, hypertension, and diabetes mellitus in the breast cancer population [7], but is increased exponentially by Cancer Treatment-Related Cardiac Dysfunction (CTRCD), particularly in patients with Her2-positive breast cancer exposed to multimodal cancer therapy.

CTRCD refers to the cardiotoxic effects of cancer treatment which can occur acutely during cancer treatment, disrupting the treatment course, or as a late effect which can adversely affect quality of life and overall survival [8]. The combination of cytotoxic chemotherapy, radiotherapy, and Anti-HER2 therapy (Trastuzumab) administered to patients with HER2 -positive breast cancer poses a significant cardiotoxic risk, particularly of left ventricular dysfunction and heart failure [8]. These risks are even higher in an ageing breast cancer population in whom pre-existing HF is increasingly common [9] and several large prospective analyses describe older patients performing worse than their younger counterparts, with cardiopulmonary disease contributing to poorer outcomes [10, 11]. The aetiology of cardiotoxicity varies amongst the most commonly used systemic agents: Trastuzumab appears to be related to impairment of HER2 signalling within myocytes [12], anthracyclines have been associated with oxidative stress and DNA damage [13], alkylating agents can induce myocardial depression and vasospasm via several pathways [14], and lastly tyrosine kinase inhibitors cause toxicity through “off target” effects [15]. Radiotherapy conversely has several described factors contributing to toxicity: DNA degradation, oxidative stress, cytokine-induced fibrosis of myocardium, pericardium, vasculature, and valvular structures [16].

Management of these patients requires a careful balance of adequate and appropriate cancer treatment while minimising cardiac risk and there is evidence that early diagnosis and treatment of CTRCD increase the potential for minimising or reversing cardiotoxic effects [17]. This emerging area of medicine has limited prospective evidence on which to base decision-making; however, the European Society of Cardiology (ESC) have recently published guidelines which aim to assist healthcare professionals providing care to oncology patients before, during and after cancer treatment with respect to their cardiovascular health and wellness [18].

The Irish National Cancer Strategy 2017–2026 [19] recommends that models of care be developed to ensure that patients receive the required care from an expert multidisciplinary clinical team. Furthermore, the 2019 Irish National Cancer Survivorship Needs Assessment identified the impact of treatment side effects as an unmet research need [20]. Indeed the European Society of Cardiology state in the 2022 Cardio-Oncology guidelines [18] list within gaps in the evidence “Robust evidence on the impact of dedicated cardio-oncology programmes”. The Understanding CARdiac Events in Breast Cancer (UCARE) study addresses this gap in current practice by developing and pilot testing a structured assessment and monitoring care pathway at Galway University Hospital for patients at risk of developing cardiac toxicity from cancer treatment.

### Study rationale

This prospective multi-centre *single-arm* trial is being done to find out if it is feasible to run a Cardio-Oncology Assessment and surveillance clinic in Galway for women with a recent diagnosis of breast cancer who will be receiving systemic chemotherapy as part of their treatment. The investigators will explore if a dedicated care pathway incorporating a comprehensive assessment of cardiac health before and during chemotherapy treatment will enable identification of those at higher risk of developing CTRCD. It is envisaged that earlier identification of high risk patients will allow protective strategies and intensive surveillance to be put in place to reduce risk and improve outcomes for these patients. This study has been assigned the ID NCT05921279 with clinicaltrials.gov.

The anticipated benefits from this research are as follows:

- This pilot feasibility study will inform specific uncertainties including practicality of delivering the intervention (Cardio-Oncology assessment and surveillance pathway) in the existing clinical setting i.e. identifying facilitators and barriers for the intervention.
- Participation in this research will provide patients with more education on their cardiac risk during cancer treatment than if they did not participate.
- Patients will be risk stratified for CTRCD at baseline which may trigger referral to cardiac specialists or increased surveillance and earlier detection of CTRCD.
- Service development and service enhancement for cancer survivors: It is expected that the projects’ output will include evidence to support the value of establishing dedicated cardio-oncology clinics and the significant role that advanced nurse practitioners/allied health professionals can play in cancer survivorship. Output will include the development of a cardio-oncology nurse specialist role.
- Educational programme development: We will incorporate our results in clinical and academic programmes. The development of a Cardio-oncology fellowship programme at Galway University Hospital, training the next generation of cardiologists to ensure that there is specialist expertise for the cardiovascular care of cancer patients.

### Study objectives

#### Primary objective

To evaluate the feasibility of implementing the intervention of a dedicated Cardio-Oncology assessment and surveillance pathway incorporating novel biomarkers, radiomic surveillance, and monitoring approaches for CTRCD into routine care for breast cancer patients undergoing adjuvant systemic chemotherapy.

#### Secondary objectives

1. To evaluate the baseline cardiovascular health of patients undergoing multimodal treatment for breast cancer, including systemic chemotherapy.
2. To identify risk factors for the development of acute CTRCD.
3. To describe the incidence of acute CTRCD in patients receiving systemic breast cancer treatment.
4. To elucidate the role of specific imaging, clinical and laboratory markers in the prediction and early detection of CTRCD in patients.
5. To collect and biobank relevant specimens and data for biomarker discovery
6. To evaluate the baseline physical activity and quality of life (QOL) in patients referred for systemic chemotherapy and the QOL implications of CTRCD.
7. To assess mental health including stress/anxiety via wearable devices in patients with high risk for CTRCT receiving systemic chemotherapy.
8. To assess patient satisfaction of the Cardio-oncology pathway and to determine their perceptions of the pathway’s usability.

Outcome measures are available in following Tables 1 and 2.

**Table 1:** Primary outcome measures

Outcome measure	Measure description	Time frame
The number of participants with successful application of guideline-directed Cardio-Oncology assessments and surveillance	To calculate the percentage of patients who successfully completed all guideline-required investigations for baseline assessments, during and post chemotherapy surveillance i.e. Echocardiography, electrocardiogram (ECG) and Cardiac biomarkers (Cardiac Troponin I/T and BNP/nt-proBNP)	2 Years

**Table 2:** Secondary outcome measures

Outcome measure	Measure description	Time frame (months)
The number of participants with cardiovascular disease amongst patients with breast cancer prior to commencement of systemic chemotherapy	To assess the incidence of cardiovascular disease at baseline	Baseline
Incidence of CTRCD in Irish breast cancer patients receiving chemotherapy	To assess the incidence of CTRCD at all post-therapy timepoints	3M, 6M, 9M, 12M, 24M
The number of participants with successful collection and biobanking specimens amongst patients with breast cancer undergoing systemic chemotherapy	To collect and biobank relevant samples	Baseline, 3M, 6M, 9M, 12M, 24M
The number of participants with successful collection of guideline-required imaging data amongst patients with breast cancer undergoing systemic chemotherapy	Feasibility of collection of guideline-required imaging data, defined as the number of participants with successful collection of guidelines-required clinical data amongst patients with breast cancer undergoing systemic chemotherapy	Baseline, 3M, 6M, 9M, 12M, 24M
The number of participants with successful collection of guideline-required clinical data amongst patients with breast cancer undergoing systemic chemotherapy	Feasibility of collection of guideline-required clinical data, defined as the number of participants with successful collection of guidelines-required clinical data amongst patients with breast cancer undergoing systemic chemotherapy	Baseline, 3M, 6M, 9M, 12M, 24M
The number of participants with common risk factors for CTRCD amongst patients with breast cancer prior to commencement of systemic chemotherapy	Using the Heart Failure Association – International Cardio-Oncology Society risk assessment tool	Baseline

### Methods

This study is a prospective, single-arm, pilot feasibility study. The study will focus on adult female patients diagnosed with stage I-III breast cancer. It will take place over multiple sites: Galway University Hospital, Sligo University Hospital, and Mayo University Hospital, led by local physicians with the aid of dedicated research nurse specialists and advanced nurse practitioners.

### Enrolment

During the initial 12 months of the study, adult female patients diagnosed with stage I–III breast cancer and referred for systemic chemotherapy will be enrolled at the time of their clinic visit for discussion and scheduling of systemic therapy.

#### Inclusion criteria

- Women aged ≥ 18 years.
- Ability to read and understand English.
- Breast Cancer Stage I–III planned to receive systemic chemotherapy.

#### Exclusion criteria

- Patients who are not for systemic chemotherapy with curative intent.
- Patients who are unable to co-operate with the study protocol.
- Patients who are unable to give informed consent.

### Consent

Patients will be consented by a member of the study team (cardio-oncology research nurse). The patient information leaflet (PIL) and informed consent form (ICF) can be found within the supplemental material. This may occur in person, as they present for their clinic visit. Alternatively, this may involve a remote consenting process (e.g. pending geographic or illness-related barriers) as follows:

- The potential participant will receive a hard copy of the PIL/ICF by email or post.
- A member of the study team will telephone the potential participant (or may use online communication platform), going through the PIL and answering any questions which participant may have.
- If the patient is happy to take part, the researcher will ask them to sign and date the ICF and send it back to the study team by email or by post.
- The member of the study who explained the study to the patient will then sign and date the ICF.
- The completed ICF (now signed and dated by both participant and research team member) will be filed in the study file which is kept securely. One copy will be placed in the patients’ medical notes and one copy will be emailed or posted back to the participant for their own records.

All enrolled patients will receive a cardiovascular assessment at baseline as per the 2022 ESC guidelines [18]. There are 4 study group cohorts amongst the study (see Table 3).

**Table 3:** Risk cohorts as stratified by HFA-ICOS cardiac risk assessment tool

Group	Cancer therapy	Risk
1	Anthracycline-based chemotherapy	Low and moderate risk
2	Anthracycline-based chemotherapy	High and very high risk
3	Herceptin-targeted therapy	Low and moderate risk
4	Herceptin-targeted therapy	High and very high risk

Each cohort will be monitored by collecting biobank bloods, ECG and transthoracic echocardiography (TTE) as per the ESC guidelines whilst receiving chemotherapy. The specific protocol for each group can be found in the supplementary material. Risk stratification will be via the Heart Failure Association – —International Cardio-Oncology Society (HFA-ICOS) cardiac risk assessment tool [21]. If consented, correspondence will be sent to the patients General Practitioner with a brief description of the study outline (see supplemental material). Correspondence will be sent to the primary medical oncologist treating the patient, with a description of their risk category and pathway selection (see supplemental material).

### Cardiac risk assessment and surveillance

Surveillance protocols will be led by the ESC 2022 guidelines in cardio-oncology across the 4 included groups (see supplementary materials). All patients will receive a comprehensive physical examination and history at follow-up visits, and be requested to complete the patient-reported outcome surveys below. Patients who in whom CTRCD is identified during cardiac surveillance on this pathway will be referred and managed appropriately as per ESC guidelines and current best practice.

Regarding long-term follow-up after the study is completed, the health of the participants will be monitored as per current best practice guidelines by the attending medical oncology follow-up clinics as per standard practice. Those patients who require dedicated cardiac follow-up will be referred accordingly as per best practice ESC guidelines.

### Questionnaires

Patients will be emailed a link to the EORTC QLQ-BR45, QLQ-30, IPAQ-SF, KCCQ-12, PSS-10 and STAI questionnaires at 3 monthly intervals over the course of 24-month period. These surveys will be accessible through any standard internet browser. Patient-Reported Quality of Life Outcomes will be collected using an electronic data capture system (REDCap). If no internet access is available, a research member team will follow up with the patient via telephone/postage system**.**

Additionally, patients, healthcare professionals and the team involved in the development and implementation of the pathway will be invited to attend either a focus group, or one to one interview, that will aim to assess experiences of the cardio-oncology care pathway, associated processes, limitations, challenges and possible benefits or recommended changes to the pathway. The patient-reported outcomes will include the following (which can be found in the supplementary material):

#### The European Organisation for research and treatment of cancer (EORTC) QLQ-C30 and QLQ BR 45

The QLQ-C30 comprises 30 items that can be summarized in 15 scales: physical functioning, role functioning, social functioning, emotional functioning, cognitive functioning, global QOL, fatigue, pain, nausea/vomiting, appetite loss, dyspnoea, sleep disturbances, diarrhoea, constipation, and financial impact of disease. Overall, the design is to measure to assess QOL amongst cancer patients [22].

Disease-specific details will be collected using EORTC modules for breast i.e. QLQ BR 45**.** This questionnaire incorporates nine multi-item scales to assess body image, sexual functioning, breast satisfaction, systemic therapy side effects, arm symptoms, breast symptoms, endocrine therapy symptoms and endocrine sexual symptoms. In addition, single items assess sexual enjoyment, future perspective and being upset by hair loss.

#### International physical activity questionnaire short form (IPAQ-SF)

IPAQ-SF assesses physical activity undertaken across a comprehensive set of domains. The specific types of activity that are assessed are walking, moderate-intensity activities and vigorous intensity activities. The frequency (measured in days per week) and duration (time per day) are collected separately for each specific type of activity. This measure assesses the types of intensity of physical activity and sitting time that people do as part of their daily lives, and can be considered to estimate total physical activity in METS per time unit, and lastly time spent sitting.

#### Kansas city cardiomyopathy questionnaire (KCCQ-12)

KCCQ was designed with input from patients and clinicians to capture those domains of how heart failure affects patients’ lives. The original KCCQ includes 23 items that map to 7 domains: symptom frequency; symptom burden; symptom stability; physical limitations; social limitations; quality of life and self-efficacy (the patient’s understanding of how to manage their heart failure). To make the KCCQ more feasible to implement in routine clinical care, it was reduced from its original 23 items (KCCQ-23) into a 12-item instrument (KCCQ-12), which includes the symptom frequency, physical limitations, social limitations and quality of life domains and can also generate the clinical and overall summary scores with excellent concordance to the respective scores of the full instrument. Use of the 12-item questionnaire will reduce the response burden for patients.

#### Perceived stress scale (PSS-10)

Stress and anxiety will be assessed subjectively using the PSS-10. Over a 10-item questionnaire, it evaluates the degree to which an individual has perceived life as unpredictable, uncontrollable and overloading over the previous month. Participants fill out the questionnaire by rating the questions about their feelings and thoughts. The total score varies from 0 (no stress) to 40 (highest stress).

#### State trait anxiety inventory (STAI)

This is a psychological inventory consisting of 40 self-report items on a 4-point Likert scale. The STAI measures two types of anxiety—state anxiety and trait anxiety. Higher scores are positively correlated with higher levels of anxiety.

#### Usability and satisfaction analysis

In order to gather information on the acceptability and satisfaction with the cardio-oncology assessment and surveillance pathway, the opinions of patients will be obtained using a satisfaction survey. It is a Likert scale questionnaire evaluating seven dimensions of patient satisfaction including general satisfaction, technical quality, interpersonal manner, communication, financial aspects, time spent with doctor and accessibility and convenience. There will also be an opportunity for patients to share their perspectives freely with a free text option.

### ECG

Baseline 12 lead ECG will be undertaken for all patients, to include measurement of QT interval (corrected). An abnormal baseline ECG will trigger referral to a cardiologist. A Stress ECG will also be undertaken in patients when clinically indicated.

### Biosensors

Machine learning and stress monitoring is an area of translational research [23]. Those patients identified in the baseline risk assessment as being at high risk of developing CTRCD will be offered wearable biosensors which will measure physiologic indicators of stress such as heart rate and blood pressure. The data from these wearable monitors (Huawei manufacture) will be correlated with chemical stress parameters (cortisol) and subjective assessments of stress in an effort to determine if the sensing technologies would be a feasible component of a future approach to monitoring stress which would facilitate early detection and intervention to prevent the progression of stress-aggravated cardiac conditions.

### Biomarkers

The schedule of biomarker sampling for these patients is outlined in the group protocols. Biomarker evaluation will be via serum/plasma/whole blood collection, and will be drawn at the time of the patient’s clinic visit for cardiac risk assessment. This will include baseline measurement of Troponin I or Troponin T (based on local laboratory availability), and NT-proBNP or BNP (again based on laboratory availability). Samples will also be biobanked for future analysis of novel markers of CTRCD risk. The custodian of these samples will be the Cancer Biobank of Department of Surgery, Lambe Institute, University of Galway.

Patients deemed to be at high risk of CTRCD will also be asked to provide a sample of saliva for measurement of salivary cortisol at these time-points. This will then be correlated with other physiological parameters of stress measured using wearable sensors, and subjective measures of stress using questionnaires. Biomarker evaluation will also be requested in the event of an unexpected hospital admission, unplanned interruption in treatment or a cardiac event.

### Cardiac imaging

TTE will be performed at baseline to inform risk stratification by providing quantitative assessment of biventricular size and function, the presence of hypertrophy, regional wall motion abnormalities, diastolic function, atrial size, valvular heart disease, septal defects, pulmonary arterial pressure, assessment of the great vessels and regarding any pericardial disease. The components of the baseline echocardiography study will be 2D and 3D image acquisition, global longitudinal strain, colour flow doppler, tissue doppler imaging and pulsed/continuous wave doppler.

The feasibility of Cardiac Magnetic Resonance assessment of CTRCD will be sought in a subgroup of patients at high risk for cardiovascular events and those with non-interpretable or inadequate assessments on conventional ultrasound imaging. This is based on recommendation 4.1 (a) in the consensus document from European Society of Medical Oncology [24].

### Data storage

All research staff working on this project in addition to the Health Service Executive (HSE) investigators are trained in General Data Protection Regulation (GDPR) as per institutional requirement. Data collected will be specific to the study and not go beyond this. All staff including the Principal Investigator and study researchers have up to date training in Good Clinical Practice. All personal information provided for this research will be anonymized via a unique identifier, and processed only to achieve the outcome of the study. It will be stored in a secure location within the clinical research facility in Galway. Regulatory bodies may also have access for project oversight purposes. Blood samples will also be biobanked in the Cancer Biobank at University of Galway and may be utilised in studies analysing novel cardio-oncology biomarkers. These studies themselves will require project specific ethics to comply with general data protection regulations 2018.

Once the health research has been completed, the pseudo-anonymised study data are either archived as required by legislation and local procedures by the sponsor and the ISF will be filed under agreement with Galway University Hospital. Study data will be maintained confidentially in a dedicated database in a secure location. This is primarily to facilitate subsequent follow-up of patients. It will be available for review by auditors or inspectors as required.

### Potential risk

A risk assessment has been carried out, taking into account local policy. There is some level of low-level harm to be expected from taking blood samples e.g. pain, anxiety, bruising, infection, damage to local structures, excessive bleeding. Of note, patients will have study bloods taken at the same time as routine bloods and so there will be no requirement for additional phlebotomy.

Adverse events refer to any untoward event or medical occurrence that may not have a causal relationship with the treatment. This study is not evaluating medicinal interventions, therefore only the following adverse events only will be recorded:

- Cardiotoxic events/CTRCD (in accordance with 2023 ESC guideline definitions)
- Cancer re-occurrence
- Hospital admission or interruption to treatment

In order to control the risk to each participant data, recording, transmission and storage of subjects’ trial-relevant data will be performed according to local secrecy obligations, as well as national and European requirements (European Union Directive 96/46 on data protection).

For electronic medical records, the researcher will need their own individual access to the electronics systems. The staff members log in will record access. Other than the patients record, the only other location personnel data are kept is in the Investigator Site File (ISF), under the governance of the primary investigator.

### Statistical analysis

The PCORE Investigators have established collaboration with biostatisticians at the INSIGHT Science Foundation Ireland centre for data analytics within University of Galway for analysis of the multi-component dataset from UCARE. All analyses will be conducted and/or supervised by the collaborating statistician at Galway University under established quality systems and standard operating procedures, and in accordance with International Conference on Harmonization E9 Statistical Principles for Clinical Trials and E6 Good Clinical Practices.

Multivariate analysis will be performed for association between Health-related QOL measures and predictors in patients undergoing breast cancer treatment. We aim to prospectively enrol approximately 100 patients in this study. A sample size calculation has not been undertaken given that this study aims to evaluate the *feasibility* of introducing a cardio-oncology pathway for breast cancer patients. This follows established practice in pilot and feasibility studies as there are areas of uncertainty which this study will address to inform the sample size calculation of future randomized controlled trials to evaluate the efficacy of this pathway in early identification and management of CTRCD. Based on known clinical activity and number of breast cancer patients referred for chemotherapy annually, we believe 100 patients to be reasonable and achievable within the timeframe of this study.

### Declaration of results

Results will be made available through presentation at academic conferences and academic publication of results with colleagues in peer-reviewed journals. Results will also be communicated directly to key knowledge-users including the HSE and the National Cancer Control Programme (via local Medical Oncology Clinical Lead). This engagement will be critical to enable to healthcare services and policy sector make informed decisions or valuable changes to clinical practice, expenditure and/or systems development to support specialized Cardio-Oncology clinical pathways. All data are to be made available upon request.

### Supplementary Information

Below is the link to the electronic supplementary material.

Supplementary material 1 (PDF 624.2 kb)

Supplementary material 2 (PDF 517.4 kb)

Supplementary material 3 (PDF 384.0 kb)

Supplementary material 4 (PDF 499.7 kb)

Supplementary material 5 (PDF 498.2 kb)

Supplementary material 6 (PDF 221.6 kb)

Supplementary material 7 (PDF 77.4 kb)

Supplementary material 8 (PDF 44.4 kb)

Supplementary material 9 (PDF 104.2 kb)

Supplementary material 10 (PDF 55.5 kb)

## Data Availability

Enquiries about data availability should be directed to the authors.

## Abbreviations

CTRCD: Cancer Treatment-Related Cardiac Dysfunction
ESC: European Society of Cardiology
QOL: Quality of life
ECG: Electrocardiogram
PIL: Patient information leaflet
ICF: Informed consent form
TTE: Transthoracic echocardiography
HFA-ICOS: Heart Failure Association International Cardio-Oncology Society
EORTC: The European Organisation for Research and Treatment of Cancer
IPAQ-SF: International Physical Activity Questionnaire Short Form
KCCQ: Kansas City Cardiomyopathy Questionnaire
PSS-10: Perceived Stress Scale
STAI: State Trait Anxiety Inventory
HSE: Health Service Executive
GDPR: General Data Protection Regulation
ISF: Investigator site file
