# Supplementary material for: Understanding cardiac events in breast cancer (UCARE): pilot cardio-oncology assessment and surveillance pathway for breast cancer patients
Source: Breast Cancer Res Treat. 2024 Jun 26;207(2):283–91. doi: 10.1007/s10549-024-07322-w (PMC11297098; doi:10.1007/s10549-024-07322-w)
Supplement: Supplementary file 1 — Supplementary material 1 (PDF 624.2 kb) [file 10549_2024_7322_MOESM1_ESM.pdf]

Hospital Headed Paper

Patient Information Leaflet /Informed Consent Form

Study title: Understanding **CAR**diac **E**vents in Breast Cancer - Pilot Cardio-Oncology Assessment and Surveillance Pathway for Breast Cancer Patients

Short title: The UCARE Study
Study code: GUH Study Code:

Principal investigators (PI) name and title: **Professor Aoife Lowery**
PI telephone no: **091 524222**
PI or dedicated study email: **aoife.lowery@universityofgalway.ie**

Principal investigators (PI) name and title: **Professor Osama Soliman**
PI telephone no:
PI or dedicated study email: **osama.soliman@universityofgalway.ie**

You are being invited to take part in a research study to be carried out at
Galway University Hospital and the University of Galway
by: The Precision Cardio Oncology Research Enterprise Team
Prof Aoife Lowery
Prof Osama Soliman
Prof Michael Kerin
Prof William Wijns

In collaboration with
Research Team at Sligo University Hospital: Dr Michael Martin, Ms Margaret Burke, Ms Moira Maxwell
and
Researchers at Mayo University Hospital : Advanced Nurse Practitioners Ms Mary Hannigan and Ms Deirdre Allen

Before you decide whether or not you wish to take part, you should read the information provided below carefully and, if you wish, discuss it with your family, friends or doctor. Take time to ask questions –you should not feel rushed or under pressure to make a quick decision.

You should clearly understand the risks and benefits of taking part in this study so that you can make a decision that is right for you. This process is known as 'Informed Consent'.

You are not under any obligation to take part in this study. If you decide not to take part, it won't affect your current or future medical care.

You can change your mind about taking part in the study at any time you like. Even if the study has started, you can still opt out. You don't have to give a reason. If you do opt out, it won't affect the quality of the medical care you get now or in the future.

Why is this study being done?

This study is being done to find out if it is feasible to run a Cardio-Oncology Assessment and Surveillance Clinic in Galway for women with a recent diagnosis of breast cancer who will be receiving systemic chemotherapy as part of their treatment. We want to explore if a dedicated care pathway incorporating a comprehensive assessment of cardiac health before and during chemotherapy treatment will enable identification of those at higher risk of developing Cancer Treatment Related Cardiac Dysfunction (CTRCD), which results from the toxic effects of chemotherapy on the heart which can lead to heart failure. It is envisaged that early identification of high risk patients will allow protective strategies and intensive surveillance to be put in place to reduce risk and improve outcomes for these patients.

The National Cancer Strategy 2017 – 2026 recommends that models of care be developed to ensure that patients receive the required care from an expert multidisciplinary clinical team. Furthermore, the 2019 National Cancer Survivorship Needs Assessment identified the impact of treatment side-effects as an unmet research need. The UCARE study addresses these needs by developing and pilot testing a structured Care Pathway at Galway University Hospital for patients at risk of developing cardiac toxicity from cancer treatment.

Who is organising and funding this study?

This study is being sponsored by the University of Galway and is being carried out in Galway by researchers from Galway University Hospital and the University of Galway in collaboration with colleagues from Mayo and Sligo University Hospitals. . The study is being funded by the Irish Cancer Society, the National Breast Cancer Research Institute and Science Foundation Ireland and is supported by the HRB-Clinical Research Facility in Galway.

Why am I being asked to take part?

You are being asked to take part because you have recently been diagnosed with breast cancer and you have been referred for chemotherapy as a component of your breast cancer treatment. A thorough assessment of heart health prior to commencement of cancer treatment enables identification of those at risk of developing cardiac side effects during treatment. For patients at

higher risk, more frequent assessment of heart function during treatment may be required so that any toxic effects of treatment on the heart can be identified and managed early.

How will the study be carried out?

The study will be carried out over 12 months and approximately 100 people will take part in the study.

Patients attending the breast clinic who are being scheduled receive chemotherapy will be invited to participate in the study.. Depending on the type of cancer treatment and their pre-treatment heart health status, patients will undergo cardiac surveillance including a questionnaire about your cardiac health, clinical examination, blood and/or saliva tests and imaging of their heart at specific timepoints during treatment. They will also follow usual care with their Oncology team. Questionnaires will also be completed at varying time points as described below over a 12-month period. You may be contacted during the study period by a research nurse, and if you have cardiac symptoms that need to be addressed, this will be managed with the assistance of your oncologist and referral to specialist cardiologists if required.

What will happen to me if I agree to take part?

If you decide to take part in the study, and sign the informed consent form (in person or remotely), the study team will review your hospital chart for information relevant to the study including your use of services at Galway University Hospital and your health outcomes. You will be assigned a unique study identification number. The data collected for the study will be collected using this unique study identification number – this is called pseudonymization. Only the Investigator and relevant people in the study team will have the 'key' to link your identity to the study number.

You will be invited to attend for a Cardiac/Heart Health Assessment visit at the start of the study at your hospital (Galway, Sligo or Mayo University Hospital) (or virtually/at an off-site location if required), where you will meet with a research nurse with expertise in heart health. The entire visit may take 2-3 hours of your time, and this time may be split across locations depending on what cardiac investigations you might need. You will review details of your medical history, cancer history and treatment with the nurse, fill in questionnaires about cardiac health and quality of life, have blood and saliva tests, a physical assessment, an ECG (tracing of the heart) and possibly an Echocardiogram (ultrasound of the heart) and receive educational materials and information relating to heart health and risks during cancer treatment.

After the initial visit, you will continue to be seen as per standard of care guidelines by your oncologists in the clinic. You may also be scheduled for further cardiac assessment and surveillance during cancer treatment, depending on your risk of developing cardiac side effects during treatment. If needed, you may also be offered closer and ambulatory cardiac monitoring using dedicated watches that measure how your heart reacts during daily life. You will be asked to complete questionnaires at the start of the study and at 3 monthly intervals during treatment. A link to the questionnaires will be emailed to you and you can complete them in your own time, on your smart phone, tablet or personal computer. At any time during the study, you may contact your clinical team (nurse, oncologist etc) as you would usually do.

You will be asked if you wish to take part in either a focus group interview OR a one to one interview, where you will be asked about your experiences of participating in the Cardio Oncology Clinical Pathway Pilot Study. The duration of the interview will be less than 60 minutes. There will be up to eight people in each focus group. Focus group interviews and one to one interview will take place at Galway University Hospital or virtually if necessary. We will talk about what worked well about the pilot study and what might not have worked so well.

The interviewer may take some notes during the discussion. All the study documents will be securely stored within the premises of Galway University Hospital and all the collected data will be kept confidential.

What are the Benefits and Risks of taking part?

Benefit: We hope that all patients in this research will benefit from more education about their heart health and potential side effects of cancer treatment. Depending on your baseline cardiac assessment, you may have more frequent cardiac assessment during treatment or earlier referral to a specialist or other expert to manage side effects if they arise. Your participation may also help other people with the same condition in the future as we learn about the needs and symptoms of women receiving cancer treatment.

Risks: The only risk we envisage is of a data breach of your data or recorded interview or focus group (if you decide to take part in an interview or focus group). The study team and Galway University Hospital take precautions to ensure that your data is kept safe. All the study documents will be securely stored within the premises of Galway University Hospital and all the collected data will be kept confidential. If you have difficulties answering the questions or are struggling with symptoms, you will be able to contact the research team.

What if something goes wrong when I am taking part in this study?

The lead research site, University Hospital Galway, has appropriate insurance in place if you are harmed as a result of participation in this trial. The risk of this happening is very low as there are no

interventions in this study other than those recommended by best practice international guidelines for monitoring heart health during cancer treatment. If you are harmed and this is due to someone's negligence then you may have grounds for a legal action for compensation against the hospital where you are being treated. The normal Health Service Executive complaints mechanisms will be available to you if appropriate.

Will it cost me anything to take part?

It will not cost you anything to take part in the study.

Will my data be kept confidential?

Yes. The research team will follow ICH GCP, a code of Good Clinical Practice in Research Conduct and the General Data Protection Regulation (GDPR) when handling your data. Information that identifies you will be kept strictly confidential. A study-specific code will instead serve as a unique identifier (pseudonymization) that will be stored separately to the main study database. The study PI may use this code to allow your oncology team to contact you should you have difficulties handling symptoms during the study. Your name will not be published or disclosed to anyone. No identifying material that can be directly linked to your records will be used in any reports or publication arising from this study.

With your consent, we will notify your GP of your participation in this study.

Records for the study will be kept in a study specific database under supervision of the HRB-Clinical Research Facility in Galway and/or in a locked filing cabinet in Galway University Hospital. The investigator and Researchers working on the study will have access to your study data. You may be contacted by the study team if an alert is triggered that suggest you need your symptoms better managed. Authorised staff, who work for University College Galway and Galway University Hospital (HSE) may require access to your details or study records to ensure that the study is being conducted in accordance with relevant regulations and Irish law.

What will happen to the blood samples?

Some of the blood samples will be sent to the laboratory for immediate analysis to check for markers of heart health, the results of these tests will be used to help determine how much cardiac assessment and surveillance you will need during cancer treatment. Some of the blood will also be stored at the Cancer Biobank located in University of Galway (https://www.universityofgalway.ie/biobank/). This is a large repository of biological samples like blood to be collected from volunteers and research participants like you. In addition some limited personal details and clinical information such as age, family history of cancer, and type of cancer are also recorded with a unique identifier to pseudonymise your identity. Your data and specimens will

be collected and stored as part of the Biobank and used solely for cancer research studies. The Biobank has had a Data Protection Impact Assessment performed in order to help safeguard your information and will only process data that is necessary to achieve the objective of any health research conducted.

Future Research Studies

We may use the data collected for use in future research in order to learn more about women's experiences such that we can learn more regarding the challenges faced by patients during and after cancer treatment. Additionally, the blood samples stored in the biobank may be used for future research to evaluate novel markers of cancer and cardiac health which will better predict how disease develops, progresses and responds to a variety of treatments.

What will happen to the results of the study?

The results of the study will be anonymised. Study information will be reported in aggregate and may be published in a peer reviewed medical journals and used for medical presentations or conferences. However, neither you nor your information will be identifiable in any report that arises from the study. If you wish to receive aggregate study results, we will send them to you after they have been published.

Who has reviewed this study?

All patient focused research in Ireland is reviewed by an independent group of people called a Research Ethics Committee, to protect your interests. This study has been reviewed by Galway Research Ethics Committee (CREC) of the Galway Teaching Hospitals. The CREC can be contacted at Merlin Park Hospital, Galway and by telephone no: 091524222

Where can I get more information?

If you need any further information about the study now or in the future, please contact:

UCARE Cardio-Oncology Study

Galway University Hospital **Email:** guh.whi@hse.ie

Clinical Studies Data Protection Notice

The SAOLTA hospitals we treat your privacy seriously. Any personal data which you provide will be treated with the highest standards of security and confidentiality, in accordance with Irish and European Data Protection legislation. This notice sets out details of the information that we collect, how we process it and who we share it with. It also explains your rights under data protection law in relation to our processing of your data.

Who we are

Throughout this Notice, “we”, “us” and “our” refers to SAOLTA University Hospital.

How we will use your personal data

By participating in this study, information about you (also called “personal data”) will be accessed, collected and stored for the purposes mentioned in the Patient Information Leaflet relating to the study. This personal data includes

- Information that directly identifies you (such as your name and your year of birth);
- Your gender, ethnic and racial background;
- Information on your health and medical condition including your medical history;
- Your treatments and your response to treatments;
- Information contained in your blood and saliva samples and the results after analysis;
- Information from your medical records.

Personal data collected at any time during the study will be kept strictly confidential. The data will be held securely at the hospital, referred to in this notice as “the clinical site”. To ensure confidentiality, the data generated during the study is **coded** with a number that will identify you in the study. Any information that leaves the clinical site will be labelled with your code instead of your name. Every person that has access to your uncoded data at the clinical site is subject to professional secrecy and confidentiality.

Data that directly identifies you (uncoded data) is stored in your medical files at the clinical site. A list or ‘key’ linking your study number to your name will also be kept securely (in a locked cupboard in a room with restricted access) by the researcher.

Who will access my personal data?

Your uncoded data will only be accessible to clinical site employees, the study researcher and site staff and the Clinical Research Ethics Committee of the Galway Teaching Hospitals (CREC) so that they can check if the study is being conducted to the best standards.

Results of the study will be provided to the ethics committee approving the study in compliance with national and international regulations on clinical studies.

Cross-border data transfers (If applicable)

In the course of processing your personal data, it may be transferred and stored on a secure database in a European country and subject to data protections as set out under the General Data Protection Regulation (GDPR). It may be transferred outside of the European Economic Area on the understanding that we rely on legally approved mechanisms to lawfully transfer data across borders including the Standard Contractual Clauses approved by the European Commission.

The purpose and legal basis for collecting your data

Any personal data you provide to us during the course of this study will be processed fairly and lawfully. Signing the Informed Consent Form means that your personal data and biological samples will be used for the purposes outlined in the Patient information leaflet (PIL). You are entitled to withdraw your consent at any time.

Personal data collected during this study and the results of the study may be presented for scientific purposes. However, you will never be identified individually during these presentations. Your identity will not be revealed in any reports or publications.

The clinical site staff, the study researcher and the members of the study's team will use your personal data within the scope defined above. If the clinical site staff, study researcher or study team wish to use your data for a purpose other the purpose specified, the researcher must contact you again to give you more information and ask your permission to use your data for the new purpose.

The General Data Protection Regulation (GDPR) allows us to process your data because the research is of substantial public interest (Articles 6(1) (e) and 9(2) (g) of the GDPR). If you require further information on the legal basis for processing your personal data, please contact Galway University Hospital's Data Protection Officer – details below.

How long we will keep your data

The personal data collected in the study will be kept for a minimum of 10 years as per GUH Code of Research Conduct after the end of the study. Thereafter, the data may be stored for a further period of time for legal reasons (e.g. revised retention obligations), or more if required by law.

Your rights

You have various rights under data protection law, subject to certain exemptions, in connection with our processing of your personal data, including the right:

- To find out if we use your personal data, access your personal data and receive copies of your personal data;
- To have inaccurate/incomplete information corrected and updated;
- In certain circumstances, to have your details deleted from systems that we use to process your personal data or have the use of your personal data restricted in certain ways;
- To object to certain processing of your data by Galway University Hospital;
- To exercise your right to data portability where applicable (i.e. obtain a copy of your personal data in a commonly used electronic form);
- To withdraw your consent to the processing of your data at any time without giving a reason by notifying your decision to the study researcher. If you withdraw your consent for data processing, your participation in the study stops and no further data will be collected from you. Your study Researcher will present you the options you have concerning your personal data;
- If, for any reason, you stop attending the study visits, your study Researcher may decide to withdraw you from the study. If this happens, we will continue to hold your data for research purposes unless you inform us that you do not want us to hold your data any longer;
- Along with study withdrawal, you have the right to request the deletion of data about you if your data are no longer necessary for the purposes of processing or there is no other legal ground for their further processing.

If you wish to exercise any of these rights, please address your request to the study researcher or the Data Protection Officer, Galway University Hospital (details below).

Questions or Complaints

If you have any questions in relation to this study please contact the study researcher or a member of the study team on guh.whi@hse.ie

Galway University Hospital is the Data Controller for research related data for research project.

If you have any complaints in connection with our processing of your personal data, you can contact GUH's Data Protection Officer (DPO): **E-mail: ddpo.west@hse.ie**

You also have the right to lodge a complaint with the Data Protection Commission if you are unhappy with our processing of your personal data. Details of how to lodge a complaint can be

found on the Data Protection Commission's website (www.dataprotection.ie), or by telephoning 1890 252 231.

Hospital Headed Paper

PATIENT CONSENT FORM

Study title: **U**nderstanding **CAR**diac **E**vents in Breast Cancer - Pilot Cardio-Oncology Assessment and Surveillance Pathway for Breast Cancer Patients

Short title: The UCARE Study **Study code:** GUH **Study Code:**

	**Initials**
I have read and understood the **Information Leaflet** about this study. The information has been fully explained to me and I have been able to ask questions, all of which have been answered to my satisfaction.	
I understand that I do not have to take part in this study and that I can opt out at any time. I understand that I do not have to give a reason for opting out and I understand that opting out will not affect my future medical care.	
I give permission for researchers to look at my medical records to get information. I have been assured that information about me will be kept private and confidential.	
I give permission for my GP to be informed of my participation in this study.	
I have been given a copy of the Information Leaflet and I will be given a copy of this completed consent form for my records.	
I consent to take part in this research study having been fully informed of the risks, benefits and alternatives.	
I give informed explicit consent to have my data processed as part of this research study.	
I give informed explicit consent to have my data processed for future research projects in the area of cancer survivorship and cancer/cardiac research.	
I understand that by giving my explicit consent to biobank my blood/saliva samples and data, I consent to them being used in cancer research (including genetic research)	
I understand that cancer research may involve the sharing of my biological samples and pseudonymised data with other research institutions	
I understand that individual research results will not be given to me and all research findings are published in the scientific literature	
I consent to be contacted by researchers as part of this research study.<br>I understand that I will be asked to give an email address and I will receive emails	

Hospital Headed Paper

with links to the questionnaires. The researchers may also send me reminders via email to complete the questionnaires.	

**If you are happy to take part in a one to one interview OR a focus group interview (in person or remotely) please choose A or B below (note you do not have to take part in either).**<br>These interviews will take place at the end of the pilot study in approximately 12 months' time:	
A.<br>I consent to take part in a one to one interview with researchers about my experience in the study.	
B.<br>I consent to take part in a focus group interview with researchers, other patients and health care professionals about my experience in the study.	

**FUTURE CONTACT**	
I consent to be re-contacted by researchers about possible future research **related** to cancer survivorship for which I may be eligible.	
I consent to the use of my pseudo-anonymised study data for future research in **the area of cancer research** without further consent being sought. I understand that any such future research will be subject to research ethics committee approval.	
I wish to receive aggregate study results after the study results have been published.	

______________________<br>Patient Name	________________<br>Patient Signature	___________<br>Date
_______________________<br>Researcher Name (Block Capitals)	________________<br>Researcher Signature	___________<br>Date

Three copies to be made: one for patient, one for Investigator site file and one for hospital records
