# Supplementary material for: Understanding cardiac events in breast cancer (UCARE): pilot cardio-oncology assessment and surveillance pathway for breast cancer patients
Source: Breast Cancer Res Treat. 2024 Jun 26;207(2):283–91. doi: 10.1007/s10549-024-07322-w (PMC11297098; doi:10.1007/s10549-024-07322-w)
Supplement: Supplementary file 2 — Supplementary material 2 (PDF 517.4 kb) [file 10549_2024_7322_MOESM2_ESM.pdf]

Table 1 Anthracycline chemotherapy - Low and Moderate Risk

**Timeline**	Baseline	C1D1	C2D1	C3D1	3M	C4D1	C5D1	6M	C6D1	9M	12M/3 M post treatment	24M/12M post treatment
**Procedures**												
**Screening/Eligibility**												
Informed Consent	X											
Screening/Eligibility	x											
Enrolment form	x											
**1. Medical History**<br>**2. HFA-ICOS Risk Assessment**<br>**3. Baseline Cancer Details**<br>**4. Oncological Treatment**	X											
**Demographics**												
Vital signs and BMI	x		x			x		x		x		
**Medications (CV & Other)**												
CV Medications	x		x			x		x		x		
Concomitant Medications	x		x			x		x		x		
**Cardiac Biomarkers (cTn/NP)**	x		x			x			x		x	
**Lipid Profile**	x		x			x			x		x	x
**Urea & Electrolytes**	x		x			x			x		x	x
**Biobank**												
Bloods for Biobank	x		x			x			x	x	x	x
**Baseline Measurements/Follow-Up Measurements**												
ECG	x											
TTE +/- Stress Echocardiography	x					x						x
Cardiac MRI**	x					x						x
CT scan of the chest (Cardiac, breast)****	x											
**Cardiac Toxicity & Cancer Related Events**												
Cardiac Event Form and Phone Interview					x			x		x	x	x
ePRO Quality of Life Surveys ***(**QLQ-30; QLQ-BR45; EQ-5D-5L; Perceived Stress Scale; Self-Evaluation)**	x				x			x		x	x	x

*saliva only collected in high risk patients who will be wearing smart sensors

**Cardiac MRI only in subset of patients if required based on Echocardiography findings/Cardiology opinion

***surveys include EORTC QLQ-C30, EORTC QLQ BR 45, EQ-5D-5L, Perceived Stress Scale PSS-10, State Trait Anxiety Inventory (STAI)

**** Will be done if oncologically indicated and assessed by cardiology team for calcification index

Table 2 Anthracycline based chemotherapy – High and Very High Risk

**Timeline**	**Baseline**	**C1D1**	**C2D1**	**3M**	**C3D1**	**C4D1**	**C5D1**	**6M**	**C6D1**	**9M**	**12M/3 M post treatment**	**24M/12M post treatment**
**Screening/Eligibility**												
Informed Consent	X											
Screening/Eligibility	x											
Enrolment form	x											
1. **Medical History**<br>2. **HFA-ICOS Risk Assessment**<br>3. **Baseline Cancer Details**<br>4. **Oncological T**	X											
**Demographics**												
Weight , BP and BMI	x		x			x		x		x		
**Medications (CV & Other)**												
CV Medication Review	x		x			x		x		x		
Concomitant Medications	x		x			x		x		x		
**Cardiac Biomarkers (cTn/NP)**	x	x	x		x	x	x		x		x	x
**Lipid Profile**	x		x			x			x		x	x
**Urea & Electrolytes**	x		x			x			x		x	x
**Biobank**												
Bloods for Biobank	x		x		x	x			x	x	x	x
saliva* for Biobank	x		x		x	x			x	x	x	
**Baseline Measurements/Follow-Up Measurements**												
ECG	x											
TTE +/- Stress Echocardiography	x		x			x			x		x	x
Cardiac MRI**	x		x			x			x		x	x
CT scan of the chest (Cardiac, breast)****	x											
**Cardiac Toxicity & Cancer Related Events**												
Cardiac Event Form and Phone Interview			x			x			x	x	x	x
**ePRO Quality of Life Surveys ***(QLQ-30; QLQ-BR45; EQ-5D-5L; Perceived Stress Scale; Self-Evaluation)**	x			x				x		x	x	x

*saliva only collected in high risk patients who will be wearing smart sensors
**Cardiac MRI only in subset of patients if required based on Echocardiography findings/Cardiology opinion
***surveys include EORTC QLQ-C30, EORTC QLQ BR 45, EQ-5D-5L, Perceived Stress Scale PSS-10, State Trait Anxiety Inventory (STAI)
**** Will be done if oncologically indicated and assessed by cardiology team for calcification index

Table 3 Her2 Targeted Therapy –Low and Moderate Risk

**Timeline**	Baseline	3M	6M	9M	12M	24M/12M post treatment
**Screening/Eligibility**						
Informed Consent	x					
Screening/Eligibility	X					
Enrolment form	x					
**1. Medical History**<br>**2. HFA-ICOS Risk Assessment**<br>**3. Baseline Cancer Details**<br>**4. Oncological Treatment**	X					
**Demographics**						
Weight BP and BMI	x	x	x	x	x	x
**Medications (CV & Other)**						
CV Medication Review	x	x	x	x	x	x
Other Medications	x	x	x	x	x	x
**Cardiac Biomarkers (cTn/NP)**	x	x	x	x	x	x
**Lipid Profile**	x	x	x	x	x	x
**Urea & Electrolytes**	x	x	x	x	x	x
**Biobank**						
Bloods for Biobank	x	x	x	x	x	x
**Baseline Measurements/Follow-Up Measurements**						
TTE +/- Stress Echocardiography	x	x	x	x	x	x
Cardiac MRI**	x	x	x	x	x	x
ECG	x					x
CT scan of the chest (Cardiac, breast)****	x					
**Cardiac Toxicity & Cancer Related Events**						
Cardiac Event Form and Phone Interview		x	x	x	x	x
ePRO Quality of Life Surveys ***<br>**(QLQ-30; QLQ-BR45; EQ-5D-5L; Perceived Stress Scale; Self-Evaluation)**	x	x	x	x	x	x

*saliva only collected in high risk patients who will be wearing smart sensors

**Cardiac MRI only in subset of patients if required based on Echocardiography findings/Cardiology opinion

***surveys include EORTC QLQ-C30, EORTC QLQ BR 45, EQ-5D-5L, Perceived Stress Scale PSS-10, State Trait Anxiety Inventory (STAI)

**** Will be done if oncologically indicated and assessed by cardiology team for calcification index

Table 4 Her2 Targeted Therapy – High Risk and Very High Risk

**Timeline**	Baseline	3M	6M	9M	12M	15M/3M post treatment	24M/12M post treatment
**Procedures**							
**Screening/Eligibility**							
Informed Consent	X						
Screening/Eligibility	x						
Enrollment form	x						
1. **Medical History**<br>2. **HFA-ICOS Risk Assessment**<br>3. **Baseline Cancer Details**<br>4. **Oncological Treatment**	X						
**Demographics**							
Weight BP and BMI	x	x	x	x	x		x
**Medications (CV & Other)**							
CV Medication Review	x	x	x	x	x		x
Other Medications	x	x	x	x	x		x
**Cardiac Biomarkers (cTn/NP)**	x	x	x	x	x	x	x
**Lipid Profile**	x	x	x	x	x	x	x
**Urea & Electrolytes**	x	x	x	x	x	x	x
**Biobank**							
Bloods for biobank	x	x	x	x	x	x	x
Saliva for Biobank	x	x	x	x	x	x	x
**Baseline Measurements/Follow-Up Measurements**							
TTE +/- Stress Echocardiography	x	x	x	x	x	x	x
Cardiac MRI**	x	x	x	x	x	x	x
ECG	x						
CT scan of the chest (Cardiac, breast)****	x						
**Cardiac Toxicity & Cancer Related Events**							
Cardiac Event Form and Phone Interview		x	x	x	x		x
**ePRO Quality of Life Surveys *** (QLQ-30; QLQ-BR45; EQ-5D-5L; Perceived Stress Scale; Self-Evaluation)**	x	x	x	x	x		x

*saliva only collected in high risk patients who will be wearing smart sensors

**Cardiac MRI only in subset of patients if required based on Echocardiography findings/Cardiology opinion

***surveys include EORTC QLQ-C30, EORTC QLQ BR 45, EQ-5D-5L, Perceived Stress Scale PSS-10, State Trait Anxiety Inventory (STAI)

**** Will be done if oncologically indicated and assessed by cardiology team for calcification index
