# Supplementary material for: Understanding cardiac events in breast cancer (UCARE): pilot cardio-oncology assessment and surveillance pathway for breast cancer patients
Source: Breast Cancer Res Treat. 2024 Jun 26;207(2):283–91. doi: 10.1007/s10549-024-07322-w (PMC11297098; doi:10.1007/s10549-024-07322-w)
Supplement: Supplementary file 3 — Supplementary material 3 (PDF 384.0 kb) [file 10549_2024_7322_MOESM3_ESM.pdf]

Advanced Therapies and Cancer Cluster
HRB Clinical Research Facility, Galway
Áis Taighde Chliniciúil HRB, Gaillimh

GP letter

Galway University
Hospital & National
University of Ireland
Galway

Date:
____

Subject: UCARE Study

Understanding **CAR**diac **E**vents in Breast Cancer **.**A Pilot Cardio-Oncology Assessment and Surveillance Pathway for Breast Cancer Patients.

Dear ....

I am contacting you today to inform you that .... is taking part in a research study being conducted at Galway, Mayo and Sligo University Hospitals. She has consented to you being informed.

The Irish Cancer Society and Cardio Oncology Research Group Prof William Wyns jointly fund this project.

The study endpoint is the Establishment of a Cardio-Oncology assessment and surveillance pathway for breast cancer patients undergoing adjuvant systemic chemotherapy at either Galway, Mayo or Sligo University Hospital and the assessment of the feasibility of trialling this approach for risk assessment and early detection of Cancer Treatment Related Cardiac Dysfunction (CTRCD)

Our objective is to

1. To evaluate the baseline cardiovascular health (CVH) of patients undergoing multimodal treatment for breast cancer, including systemic chemotherapy
2. To identify host and treatment related risk factors for the development of acute CTRCD in a cohort of Irish patients undergoing multimodal treatment for breast cancer
3. To describe the incidence of acute CTRCD in a cohort of Irish patients undergoing multimodal treatment for breast cancer, including systemic chemotherapy.
4. To elucidate the role of specific imaging, clinical and laboratory markers in the prediction and early detection of CTRCD in patients undergoing multimodal treatment for breast cancer

Breast Cancer patients scheduled for chemotherapy routinely have baseline Cardiac Echo and ECG before chemotherapy. In this study, we will formalise the risk assessment of patients scheduled for breast cancer treatment. Patients with higher risk will have closer monitoring by a cardiologist with more frequent cardiac echos and ECG and we will also take some blood for cardiac biomarkers

To gain a better insight into the breast cancer patients cardiac performance status post chemotherapy, we will follow up the patients for 12 months after treatment. Ultimately, we want to determine if closer monitoring will lead us to creating an improved patient pathway.

We aim to enrol 100 patients onto this study

Please feel free to contact me if you require further information.

Kind regards

____

Professor Aoife Lowery
National University of Ireland Galway.

E. aoife.lowery@nuigalway.ie
