# Supplementary material for: Understanding cardiac events in breast cancer (UCARE): pilot cardio-oncology assessment and surveillance pathway for breast cancer patients
Source: Breast Cancer Res Treat. 2024 Jun 26;207(2):283–91. doi: 10.1007/s10549-024-07322-w (PMC11297098; doi:10.1007/s10549-024-07322-w)
Supplement: Supplementary file 4 — Supplementary material 4 (PDF 499.7 kb) [file 10549_2024_7322_MOESM4_ESM.pdf]

OLLSCOIL NA GAILLIMHE
UNIVERSITY OF GALWAY

Saolta
Grúpa Ollscoile Cúram Sláinte
University Health Care Group

Study Title: UCARE - Understanding CARdiac Events in Breast Cancer .A Pilot Cardio-Oncology Assessment and Surveillance Pathway for Breast Cancer Patients as an Intervention For The Early Detection of Cancer Treatment Related Cardiac Dysfunction(CTRCD)

Patient Details: *insert patient details here*

Dear *(Insert medical oncologist details here)*

Your patient (*name & board number*) has been enrolled on the UCARE study and as part of this study has had a Heart Failure Association – International Cardio-Oncology Society (HFA-ICOS) risk assessment performed.

This letter is to inform you that the HFA-ICOS risk assessment has indicated that this patient is at HIGH RISK of developing CTRCD as a result of their planned treatment with (*insert chemotherapy/Herceptin details here).*

As part of the UCARE study they have been allocated to a high risk surveillance pathway in line with European Society of Cardiology Guidelines
https://academic.oup.com/eurheartj/article/43/41/4229/6673995

These guidelines also recommend that high risk patients have a cardiology assessment prior to commencing treatment

Please do not hesitate to contact us if any further information is required.

Kind Regards

UCARE Investigators

Prof Aoife Lowery   Prof Osama Soliman

Ollscoil na Gaillimhe,
Bóthar na hOllscoile,
Gaillimh, H91 TK33,
Éire

University of Galway,
University Road,
Galway, H91 TK33,
Ireland

Ollscoil na Gaillimhe,
Bóthar na hOllscoile,
Gaillimh, H91 TK33,
Éire

University of Galway,
University Road,
Galway, H91 TK33,
Ireland
