# Supplementary material for: Understanding cardiac events in breast cancer (UCARE): pilot cardio-oncology assessment and surveillance pathway for breast cancer patients
Source: Breast Cancer Res Treat. 2024 Jun 26;207(2):283–91. doi: 10.1007/s10549-024-07322-w (PMC11297098; doi:10.1007/s10549-024-07322-w)
Supplement: Supplementary file 7 — Supplementary material 7 (PDF 77.4 kb) [file 10549_2024_7322_MOESM7_ESM.pdf]

ENGLISH

EORTC QLQ-BR45

Patients sometimes report that they have the following symptoms or problems. Please indicate the extent to which you have experienced these symptoms or problems during the past week. Please answer by circling the number that best applies to you.

**During the past week:**		**Not at All**	**A Little**	**Quite a Bit**	**Very Much**
31.	Have you had a dry mouth?	1	2	3	4
32.	Have food and drink tasted different than usual?	1	2	3	4
33.	Have your eyes been painful, irritated or watery?	1	2	3	4
34.	Have you lost any hair?	1	2	3	4
35.	Answer this question only if you have lost any hair: Have you been upset by the loss of your hair?	1	2	3	4
36.	Have you felt ill or unwell?	1	2	3	4
37.	Have you had hot flushes?	1	2	3	4
38.	Have you had headaches?	1	2	3	4
39.	Have you felt physically less attractive as a result of your disease or treatment?	1	2	3	4
40.	Have you felt less feminine as a result of your disease or treatment?	1	2	3	4
41.	Have you had problems looking at yourself naked?	1	2	3	4
42.	Have you been dissatisfied with your body?	1	2	3	4
43.	Have you worried about your health in the future?	1	2	3	4

**During the past four weeks:**		**Not at All**	**A Little**	**Quite a Bit**	**Very Much**
44.	Have you been interested in sex?	1	2	3	4
45.	Have you been sexually active (with or without intercourse)?	1	2	3	4
46.	Has sex been enjoyable for you?	1	2	3	4

SPECIMEN

Please go on to the next page

During the past week:	Not at All	A Little	Quite a Bit	Very Much
47. Have you had any pain in your arm or shoulder?	1	2	3	4
48. Have you had a swollen arm or hand?	1	2	3	4
49. Have you had problems raising your arm or moving it sideways?	1	2	3	4
50. Have you had any pain in the area of your affected breast?	1	2	3	4
51. Has the area of your affected breast been swollen?	1	2	3	4
52. Has the area of your affected breast been oversensitive?	1	2	3	4
53. Have you had skin problems on or in the area of your affected breast (e.g., itchy, dry, flaky)?	1	2	3	4
54. Have you sweated excessively?	1	2	3	4
55. Have you had mood swings?	1	2	3	4
56. Have you been dizzy?	1	2	3	4
57. Have you had soreness in your mouth?	1	2	3	4
58. Have you had any redness in your mouth?	1	2	3	4
59. Have you had pain in your hands or feet?	1	2	3	4
60. Have you had any redness on your hands or feet?	1	2	3	4
61. Have you had tingling in your fingers or toes?	1	2	3	4
62. Have you had numbness in your fingers or toes?	1	2	3	4
63. Have you had problems with your joints?	1	2	3	4
64. Have you had stiffness in your joints?	1	2	3	4
65. Have you had pain in your joints?	1	2	3	4
66. Have you had aches or pains in your bones?	1	2	3	4
67. Have you had aches or pains in your muscles?	1	2	3	4
68. Have you gained weight?	1	2	3	4
69. Has weight gain been a problem for you?	1	2	3	4

SPECIMEN

Please go on to the next page

**During the past <u>four</u> weeks:**	**Not at All**	**A Little**	**Quite a Bit**	**Very Much**
70. Have you had a dry vagina?	1	2	3	4
71. Have you had discomfort in your vagina?	1	2	3	4

**Please answer the following two questions only if you have been sexually active:**	**Not at All**	**A Little**	**Quite a Bit**	**Very Much**
72. Have you had pain in your vagina during sexual activity?	1	2	3	4
73. Have you experienced a dry vagina during sexual activity?	1	2	3	4

**During the past week:**	**Not at All**	**A Little**	**Quite a Bit**	**Very Much**
74. Have you been satisfied with the cosmetic result of the surgery?	1	2	3	4
75. Have you been satisfied with the appearance of the skin of your affected breast (thoracic area)?	1	2	3	4

SPECIMEN

© 2018 EORTC Quality of Life Group. All right reserved. Phase IV module.
