# Supplementary material for: Understanding cardiac events in breast cancer (UCARE): pilot cardio-oncology assessment and surveillance pathway for breast cancer patients
Source: Breast Cancer Res Treat. 2024 Jun 26;207(2):283–91. doi: 10.1007/s10549-024-07322-w (PMC11297098; doi:10.1007/s10549-024-07322-w)
Supplement: Supplementary file 8 — Supplementary material 8 (PDF 44.4 kb) [file 10549_2024_7322_MOESM8_ESM.pdf]

ENGLISH

EORTC QLQ-C30 (version 3)

We are interested in some things about you and your health. Please answer all of the questions yourself by circling the number that best applies to you. There are no "right" or "wrong" answers. The information that you provide will remain strictly confidential.

Please fill in your initials:
Your birthdate (Day, Month, Year):
Today's date (Day, Month, Year): 31

		Not at All	A Little	Quite a Bit	Very Much
1.	Do you have any trouble doing strenuous activities, like carrying a heavy shopping bag or a suitcase?	1	2	3	4
2.	Do you have any trouble taking a long walk?	1	2	3	4
3.	Do you have any trouble taking a short walk outside of the house?	1	2	3	4
4.	Do you need to stay in bed or a chair during the day?	1	2	3	4
5.	Do you need help with eating, dressing, washing yourself or using the toilet?	1	2	3	4

During the past week:

		Not at All	A Little	Quite a Bit	Very Much
6.	Were you limited in doing either your work or other daily activities?	1	2	3	4
7.	Were you limited in pursuing your hobbies or other leisure time activities?	1	2	3	4
8.	Were you short of breath?	1	2	3	4
9.	Have you had pain?	1	2	3	4
10.	Did you need to rest?	1	2	3	4
11.	Have you had trouble sleeping?	1	2	3	4
12.	Have you felt weak?	1	2	3	4
13.	Have you lacked appetite?	1	2	3	4
14.	Have you felt nauseated?	1	2	3	4
15.	Have you vomited?	1	2	3	4
16.	Have you been constipated?	1	2	3	4

Please go on to the next page

During the past week:	Not at All	A Little	Quite a Bit	Very Much
17. Have you had diarrhea?	1	2	3	4
18. Were you tired?	1	2	3	4
19. Did pain interfere with your daily activities?	1	2	3	4
20. Have you had difficulty in concentrating on things, like reading a newspaper or watching television?	1	2	3	4
21. Did you feel tense?	1	2	3	4
22. Did you worry?	1	2	3	4
23. Did you feel irritable?	1	2	3	4
24. Did you feel depressed?	1	2	3	4
25. Have you had difficulty remembering things?	1	2	3	4
26. Has your physical condition or medical treatment interfered with your family life?	1	2	3	4
27. Has your physical condition or medical treatment interfered with your social activities?	1	2	3	4
28. Has your physical condition or medical treatment caused you financial difficulties?	1	2	3	4

For the following questions please circle the number between 1 and 7 that best applies to you

29. How would you rate your overall health during the past week?

1	2	3	4	5	6	7
Very poor						Excellent

30. How would you rate your overall quality of life during the past week?

1	2	3	4	5	6	7
Very poor						Excellent

© Copyright 1995 EORTC Quality of Life Group. All rights reserved. Version 3.0
