# Supplementary material for: Understanding cardiac events in breast cancer (UCARE): pilot cardio-oncology assessment and surveillance pathway for breast cancer patients
Source: Breast Cancer Res Treat. 2024 Jun 26;207(2):283–91. doi: 10.1007/s10549-024-07322-w (PMC11297098; doi:10.1007/s10549-024-07322-w)
Supplement: Supplementary file 9 — Supplementary material 9 (PDF 104.2 kb) [file 10549_2024_7322_MOESM9_ESM.pdf]

Cardiomyopathy Questionnaire (Kansas City) (KCCQ-12)

The following questions refer to your **heart failure** and how it may affect your life. Please read and complete the following questions. There are no right or wrong answers. Please mark the answer that best applies to you.

1. **Heart failure** affects different people in different ways. Some may mainly feel shortness of breath while others mainly fatigue. Please indicate how much you have been limited by **heart failure** (for example, shortness of breath or fatigue) in your ability to do the following activities over the past 2 weeks.

Please place an **X** in one box on each line

**Activity**	**Extremely** limited	**Quite a bit** limited	**Moderately** limited	**Slightly** limited	**Not at all** limited	**Limited for other reasons or did not do the activity**
Showering/Bathing	❑	❑	❑	❑	❑	❑
Walking 1 block on level ground	❑	❑	❑	❑	❑	❑
Jogging or hurrying (as if to catch a bus)	❑	❑	❑	❑	❑	❑

2. Over the past 2 weeks, how many times did you have **swelling** in your feet, ankles or legs when you woke up in the morning?

Every morning	3 or more times a week, but not every day	1-2 times a week	Less than once a week	Never over the past 2 weeks
❑	❑	❑	❑	❑

3. Over the past 2 weeks, on average, how many times has **fatigue** limited your ability to do what you wanted?

All of the time	Several times a day	At least once a day	3 or more times a week but not every day	1-2 times a week	Less than once a week	Never over the past 2 weeks
❑	❑	❑	❑	❑	❑	❑

4. Over the past 2 weeks, on average, how many times has **shortness of breath** limited your ability to do what you wanted?

All of the time	Several times a day	At least once a day	3 or more times a week but not every day	1-2 times a week	Less than once a week	Never over the past 2 weeks
❑	❑	❑	❑	❑	❑	❑

Copyright © 2012 John Spertus, MD, MPH

5. Over the past 2 weeks, on average, how many times have you been forced to sleep sitting up in a chair or with at least 3 pillows to prop you up because of **shortness of breath**?

Every night	3 or more times a week, but not every night	1-2 times a week	Less than once a week	Never over the past 2 weeks
❑	❑	❑	❑	❑

6. Over the past 2 weeks, how much has your **heart failure** limited your enjoyment of life?

It has **extremely** limited my enjoyment of life	It has limited my enjoyment of life **quite a bit**	It has **moderately** limited my enjoyment of life	It has **slightly** limited my enjoyment of life	It has **not limited** my enjoyment of life at all
❑	❑	❑	❑	❑

7. If you had to spend the rest of your life with your **heart failure** the way it is right now, how would you feel about this?

Completely dissatisfied	Mostly dissatisfied	Fairly satisfied	Mostly satisfied	Completely satisfied
❑	❑	❑	❑	❑

8. How much does your **heart failure** affect your lifestyle? Please indicate how your **heart failure** may have limited your participation in the following activities over the past 2 weeks.

Please place an **X** in one box on each line

**Activity**	**Extremely** limited	**Quite a bit** limited	**Moderately** limited	**Slightly** limited	**Not at all** limited	**Limited for other reasons or did not do the activity**
Hobbies, recreational activities	❑	❑	❑	❑	❑	❑
Working or doing household chores	❑	❑	❑	❑	❑	❑
Visiting family or friends out of your home	❑	❑	❑	❑	❑	❑

Copyright © 2012 John Spertus, MD, MPH
