# Supplementary material for: Understanding cardiac events in breast cancer (UCARE): pilot cardio-oncology assessment and surveillance pathway for breast cancer patients
Source: Breast Cancer Res Treat. 2024 Jun 26;207(2):283–91. doi: 10.1007/s10549-024-07322-w (PMC11297098; doi:10.1007/s10549-024-07322-w)
Supplement: Supplementary file 10 — Supplementary material 10 (PDF 55.5 kb) [file 10549_2024_7322_MOESM10_ESM.pdf]

Perceived Stress Scale Questionnaire 1

Participant ID: ______________________________ Data/Time: __________________________

The Perceived Stress Scale (PSS) is a classic stress assessment instrument. This tool, while originally developed in 1983, remains a popular choice for helping us understand how different situations affect our feelings and our perceived stress. The questions in this scale ask about your feelings and thoughts during the last month. In each case, you will be asked to indicate how often you felt or thought a certain way. Although some of the questions are similar, there are differences between them, and you should treat each one as a separate question. The best approach is to answer quickly. That is, don't try to count the number of times you felt a particular way; rather indicate the alternative that seems like a reasonable estimate.

For each question choose from the following alternatives:

0 – never **1 - rarely** **2 – sometimes** **3 - fairly often** **4 - very often**

1. In the last month, how often have you been upset because of something that happened unexpectedly? _________

2. In the last month, how often have you felt that you were unable to control the important things in your life? _________

3. In the last month, how often have you felt nervous and stressed? _________

4. In the last month, how often have you felt confident about your ability to handle your personal problems? _________

5. In the last month, how often have you felt that things were going your way? _________

6. In the last month, how often have you found that you could not cope with all the things that you had to do? _________

7. In the last month, how often have you been able to control irritations in your life? _________

8. In the last month, how often have you felt that you were on top of things? _________

9. In the last month, how often have you been angered because of things that happened that were outside of your control? _________

10. In the last month, how often have you felt difficulties were piling up so high that you could not overcome them? _________

Perceived Stress Scale Questionnaire 2

Participant ID: ______________________________ Data/Time: __________________________

The Perceived Stress Scale (PSS) is a classic stress assessment instrument. This tool, while originally developed in 1983, remains a popular choice for helping us understand how different situations affect our feelings and our perceived stress. The questions in this scale ask about your feelings and thoughts during the last month. In each case, you will be asked to indicate how often you felt or thought a certain way. Although some of the questions are similar, there are differences between them, and you should treat each one as a separate question. The best approach is to answer quickly. That is, don't try to count the number of times you felt a particular way; rather indicate the alternative that seems like a reasonable estimate.

For each question choose from the following alternatives:

0 – never **1 - rarely** **2 – sometimes** **3 - fairly often** **4 - very often**

1. After the experiment, how often have you been upset because of something that happened unexpectedly? _________

2. After the experiment, how often have you felt that you were unable to control the important things in your life? _________

3. After the experiment, how often have you felt nervous and stressed? _________

4. After the experiment, how often have you felt confident about your ability to handle your personal problems? _________

5. After the experiment, how often have you felt that things were going your way? _________

6. After the experiment, how often have you found that you could not cope with all the things that you had to do? _________

7. After the experiment, how often have you been able to control irritations in your life? _________

8. After the experiment, how often have you felt that you were on top of things? _________

9. After the experiment, how often have you been angered because of things that happened that were outside of your control? _________

10. After the experiment, how often have you felt difficulties were piling up so high that you could not overcome them? _________
